# Efficacy and safety of a proposed omalizumab biosimilar compared to the reference product in the management of uncontrolled moderate-to-severe allergic asthma: a multicenter, phase III, randomized, double-blind, equivalency clinical trial

**DOI:** 10.3389/fimmu.2024.1425906

**Published:** 2024-07-29

**Authors:** Mostafa Ghanei, Babak Ghalebaghi, Ramin Sami, Mehdi Torabizadeh, Majid Mirsadraee, Babak Amra, Marzieh Tavakol, Hanieh Raji, Morteza Fallahpour, Arda Kiani, Atefeh Abedini, Farahzad Jabbari Azad, Seyed Alireza Mahdaviani, Davood Attaran, Mohammad Samet, Sasan Tavana, Maryam Haddadzadeh shoushtari, Javad Nazari, FatemehAlsadat AghaeiMeybodi, Mohammad Reza Fazlollahi, Ramin Ghasemi, Araz Sabzvari, Hamidreza Kafi, Esmaeil Idani

**Affiliations:** ^1^ Chemical Injuries Research Center, Systems Biology and Poisonings Institute, Baqiyatallah University of Medical Sciences, Tehran, Iran; ^2^ Ear, Nose, Throat and Head and Neck Surgery Research Center, Iran University of Medical Sciences, Tehran, Iran; ^3^ Department of Internal Medicine, School of Medicine, Khorshid Hospital, Isfahan University of Medical Sciences, Isfahan, Iran; ^4^ Golestan Hospital Clinical Research Development Unit, Ahvaz Jundishapur University of Medical Sciences, Ahvaz, Iran; ^5^ Department of Internal Medicine, Islamic Azad University, Mashhad, Iran; ^6^ Bamdad Respiratory and Sleep Research Center, Isfahan University of Medical Sciences, Isfahan, Iran; ^7^ Non-communicable Diseases Research Center, Alborz University of Medical Sciences, Karaj, Iran; ^8^ Department of Internal Medicine, Air Pollution and Respiratory Diseases Research Center, Ahvaz Jundishapur University of Medical Sciences, Ahvaz, Iran; ^9^ Allergy Department, Rasoul Akram Hospital, Iran University of Medical Sciences, Tehran, Iran; ^10^ Tracheal Diseases Research Center, National Research Institute of Tuberculosis and Lung Diseases (NRITLD), Shahid Beheshti University of Medical Sciences and Health Services, Tehran, Iran; ^11^ Chronic Respiratory Diseases Research Center, National Research Institute of Tuberculosis and Lung Diseases (NRITLD), Shahid Beheshti University of Medical Sciences and Health Services, Tehran, Iran; ^12^ Allergy Research Center, Mashhad University of Medical Sciences, Mashhad, Iran; ^13^ Pediatric Respiratory Diseases Research Center, National Research Institute of Tuberculosis and Lung Diseases (NRITLD), Shahid Beheshti University of Medical Sciences, Tehran, Iran; ^14^ Respiratory & Critical Care Division, Mashhad University of Medical Sciences, Mashhad, Iran; ^15^ Department of Internal Medicine, Division of Pulmonology, Shahid Sadoughi University of Medical Sciences, Yazd, Iran; ^16^ Department of Pulmonary Medicine, Clinical Research and Development Center, Shahid Modarres Hospital, Shahid Beheshti University of Medical Sciences, Tehran, Iran; ^17^ Air Pollution and Respiratory Diseases Research Center, Ahvaz Jundishapur University of Medical Sciences, Ahvaz, Iran; ^18^ Lung Department, Ebnesina Hospital, Tehran, Iran; ^19^ Immunology, Asthma and Allergy Research Institute, Tehran University of Medical Sciences, Tehran, Iran; ^20^ Eisabne Maryam Hospital, Medical School, Isfahan University of Medical Sciences, Isfahan, Iran; ^21^ CinnaGen Medical Biotechnology Research Center, Alborz University of Medical Sciences, Karaj, Iran; ^22^ Medical Department, Orchid Pharmed Company, Tehran, Iran; ^23^ Pulmonary Rehabilitation Research Center (PRRC), National Research Institute of Tuberculosis and Lung Diseases (NRITLD), Shahid Beheshti University of Medical Sciences, Tehran, Iran

**Keywords:** asthma, omalizumab, biosimilar, IgE, allergic

## Abstract

**Background and aims:**

Allergic asthma has a considerable burden on the quality of life. A significant portion of moderate-to-severe allergic asthma patients need omalizumab, an anti-immunoglobulin-E monoclonal antibody, as an add-on therapy. In this phase III clinical trial P043 (Zerafil^®^, CinnaGen, Iran) efficacy, safety, and immunogenicity were compared with Xolair^®^ (the originator omalizumab). The primary outcome was the rate of protocol-defined asthma exacerbations.

**Methods:**

Exacerbation rates, Asthma Control Test (ACT) results, spirometry measurements, immunogenicity, and safety were evaluated. Each subject received either medication with a dose ranging from 150 to 375 mg based on pre-treatment serum total IgE level (IU/mL) and body weight (kg) every two or four weeks for a duration of 28 weeks.

**Results:**

Exacerbation rates were 0.150 (CI: 0.079-0.220) in the P043 group, and 0.190 (CI: 0.110-0.270) in the omalizumab group (per-protocol). The least squares mean differences of predicted Forced Expiratory Volume in the First second (FEV_1_) were -2.51% (CI: -7.17-2.15, P=0.29) and -3.87% (CI: -8.79-1.04, P=0.12), pre- and post-bronchodilator use. The mean ± SD of ACT scores at the screening and the last visit were 10.62 ± 2.93 and 20.93 ± 4.26 in P043 and 11.09 ± 2.75 and 20.46 ± 5.11 in the omalizumab group. A total of 288 adverse events were reported for the 256 enrolled participants. Among all, “dyspnea” and “headache” were the most reported ones. The overall incidence of adverse events (P=0.62) and serious adverse events (P=0.07) had no significant differences between the two groups. None of the samples were positive for anti-drug antibodies.

**Conclusion:**

P043 was equivalent to omalizumab in the management of asthma in reduction of exacerbations. There was no significant difference in other efficacy and safety parameters.

**Clinical trial registration:**

www.clinicaltrials.gov (NCT05813470) and www.IRCT.ir (IRCT20150303021315N20).

## 1 Introduction

Asthma is the result of airway inflammation and presents itself with unease of breathing. It exhibits a high prevalence, ranging from 3.3% in Iran to 10.4% in the US (2019, IHME) (1). Moderate-to-severe asthma is now controlled with biologic agents such as anti-interleukin (anti-IL) 5 or anti-immunoglobulin E (anti-IgE) drugs as add-on therapies (2). Omalizumab binds to low and high-affinity receptors (FcϵRI and FcϵRII) of IgE and thus reduces the serum concentration of free IgE. The reduction in IgE levels decreases the rate of FcϵRI expression on mast cells, dendritic cells, and basophils, resulting in lower inflammatory responses in peripheral and bronchial tissues and a decrease in IL-2, 4, 5, and 13 (3). Omalizumab use in allergic asthma is also associated with IL-25 and 33 levels reduction (4).

According to the Global Initiative for Asthma (GINA), 17% of asthmatic patients are categorized into different-to-treat class (2). Omalizumab is the first-line therapy as an add-on to inhaled corticosteroids (ICS) treatment for uncontrolled stage 4 asthma. Omalizumab use is estimated to decrease the annual rate of exacerbations by 38% (5) and reduce the need for inhaled or oral corticosteroids as well (6, 7). The need to use systemic corticosteroid bursts in omalizumab users is expected to be 43% lower than in non-biologic treatments (5). A side effect of prolonged ICS use is an elongated IgE response, which can be controlled with omalizumab use (8). It seems that omalizumab provides a protective effect on lung function in severe asthma (9). Omalizumab is also known to alleviate allergic rhinitis, a major disease burden for asthma patients (10, 11).

It is estimated that 60% of asthma costs are associated with severe, uncontrolled asthma (12). This life-challenging disorder requires affordable and effective treatment options, which justifies an equivalency clinical study for a new biosimilar of omalizumab compared to the originator brand, Xolair^®^ (2, 13). There are several studies on the efficacy and safety of omalizumab biosimilars worldwide (14). While the majority of these studies are focused on treatment options for urticaria, this study targets uncontrolled severe atopic asthma patients, for whom this medication can effectively increase the quality of life (15–20).

## 2 Methods

### 2.1 Study design and intervention

This study was a phase III, randomized, multicenter, double-blind, two-armed, parallel, equivalency clinical trial to compare the efficacy and safety of P043 (Zerafil^®^, CinnaGen, Iran) in comparison to omalizumab (Xolair^®^, Genentech, Inc., USA and Novartis Pharmaceuticals Corp, Switzerland) in patients with uncontrolled moderate-to-severe allergic asthma. Patients were randomly assigned to one of the two groups (1:1). Each patient received either P043 or omalizumab subcutaneously. The medication was administered every two or four weeks to provide a dose ranging from 150 to 375 mg of either intervention, based on each patient’s pre-treatment serum total IgE level (IU/mL) and body weight (kg) for a duration of 28 weeks.

### 2.2 Participants

The patients were between 18 to 75 years old and were diagnosed with moderate-to-severe persistent allergic asthma requiring regular treatment with a high dose of ICS (GINA 2019 step 4 treatment). The subjects had to have a total serum IgE levels of ≥30 to ≤700 IU/mL, body weight of ≥30 to ≤150 kg, and a history of one of these two items during the past 12 months: At least two asthma exacerbations that needed systemic corticosteroids, and severe asthma exacerbation in which peak expiratory flow (PEF) or forced expiratory volume in the first second (FEV_1_) was less than 60% of the patient’s best result, needing systemic corticosteroids and hospitalization or an emergency department visit. The patients were required to have the evidence of allergies to at least one perennial aeroallergen, including dog, cat, cockroach, Dermatophagoides farinae, or Dermatophagoides pteronyssinus.

The key exclusion criteria were as follows: history of an asthma exacerbation requiring intubation during the last 12 months; smoking history of ≥10 pack-years; history of chronic corticosteroid use (20 to 30 mg prednisolone for more than three weeks) or other immunosuppressants due to conditions other than asthma; history of treatment with omalizumab in the past 12 months or severe allergic or anaphylactic reactions to omalizumab; an active lung disease other than asthma; acute upper respiratory tract infection within previous month. Pregnant women or those unwilling to use proper contraception were also excluded.

All patients provided written informed consent forms prior to screening. The study protocol was approved by the ethics committees of Shahid Beheshti University of Medical Sciences (IR.SBMU.NRITLD.REC.1399.133) and Tehran University of Medical Sciences (IR.TUMS.VCR.REC.1399.580). The study was designed and conducted in accordance with the Good Clinical Practice guidelines and the Declaration of Helsinki and was registered at www.clinicaltrials.gov (NCT05813470) and www.IRCT.ir (IRCT20150303021315N20).

### 2.3 Randomization and blinding

Patients meeting the inclusion criteria were randomly assigned to different groups using a stratified randomization method. The randomization was performed using R-CRAN-version 3.2.3, using blocks of size 2. Randomization was stratified according to baseline asthma medications, including ICS + long-acting beta agonists (LABA); ICS ± other treatments (except oral corticosteroids (OCS) and LABA); ICS + LABA + other treatments (except OCS); OCS + ICS + LABA ± other treatments; and the specific type of ICS used (Fluticasone, Budesonide). The patients who were receiving oral corticosteroids prior to the study enrollment received the same dose in the course of the study and were stratified into the OCS + ICS + LABA ± Other treatment class. The other participants who were stratified into other medication classes did not receive any OCS. All participants, caregivers, and outcome assessors were blinded to the treatment allocation.

### 2.4 Outcomes

The primary outcome of the study was the rate of protocol-defined asthma exacerbations (PDAEs) during the 28-week treatment period. PDAE was defined as worsening asthma symptoms requiring treatment with 40-50 mg of oral prednisolone (or equivalent doses of other corticosteroids) for three to seven days. For patients receiving long-term oral corticosteroids, an exacerbation was defined as at least a 20-mg increase in the average daily dose of oral prednisolone. The secondary endpoints were the changes in spirometry measures (FEV_1_), safety and immunogenicity assessment, and the change in Asthma Control Test (ACT) score from baseline to the end of the trial (28 weeks). ACT scores range from 5 to 25. Scores of 20-25 are classified as well-controlled asthma; 16-19 as not well-controlled; and 5-15 as very poorly controlled asthma. The Persian ACT questionnaire was validated and its reliability was assessed previously by Sigari et al. (21).

#### 2.4.1 Safety assessment

Safety assessments were performed during the study, and all adverse events (AEs) were recorded during scheduled visits. All AEs were categorized based on preferred term (PT) and system organ class (SOC) according to medical dictionary for regulatory activities (MedDRA) terms. In addition, all reported events were graded using the national cancer institute common terminology criteria for adverse events (CTCAE) v5.0 (22). The seriousness of AEs was specified based on ICH E2B guidelines (23). Moreover, the causality assessment of the AEs was done based on the world health organization (WHO) criteria.

Since one patient in P043 group and three patients in omalizumab group were withdrawn from the study before receiving any injections, 252 patients were included in safety analysis.

The AEs of Special Interest (AESIs) included: Injection site reactions, anaphylactic reactions, hypersensitivity, vasculitis, serum sickness, transient ischemic attack (TIA), ischemic stroke, and malignant neoplasms.

#### 2.4.2 Immunogenicity

BioSim™ anti-omalizumab ELISA kit was used to assess the presence of anti-omalizumab antibodies and was validated according to International Council for Harmonization (ICH) M10 for use at the enrollment, and the 16^th^ and 28^th^ weeks.

### 2.5 Statistical analysis

In each group, 115 patients were required to achieve 80% power to detect equivalence based on the rate difference between the groups for PDAEs with a margin of error of ±0.20 and a significance level of 0.05. The 28-week rate of PDAEs in the reference group (omalizumab) in the INNOVATE phase III clinical study was 0.68 (24). A total sample size of 256 patients was calculated based on a drop-out rate of 10%.

Poisson regression models with regard to overdispersion assumption, adjusted for baseline eosinophils and dosing schedule, were used to compare the PDAE rates. Efficacy was judged equivalent if the lower and upper limits of the 95% confidence intervals (95% CIs) for differences in PDAEs were within the accepted equivalence margin (-0.2, 0.2). In the case of a premature discontinuation, the number of clinically significant asthma exacerbations was imputed. Missing values were imputed for patients who received at least one dose of study medication. Primary analysis was performed in the per-protocol (PP) and intention-to-treat (ITT) populations.

Patients with PP status were those who completed the study without major deviations from the protocol. In the ITT population, all randomized patients were included, and data were analyzed according to their study arm assignment. Secondary efficacy analyses were performed in the ITT population.

The generalized estimating equation (GEE) model was used to analyze ACT scores from baseline to the end of the 28 weeks. Analyzing changes in spirometry measures (FEV_1_) was done using the ANCOVA model. All patients who received at least one dose of the study medication, were included in the safety population. Safety analyses were conducted using descriptive statistics, and chi-squared tests were used to compare incidence rates. All the statistical analyses were conducted using STATA version 14.0 and R 3.2.3 with a significance level of 0.05 for all tests.

## 3 Results

**Figure 1:**
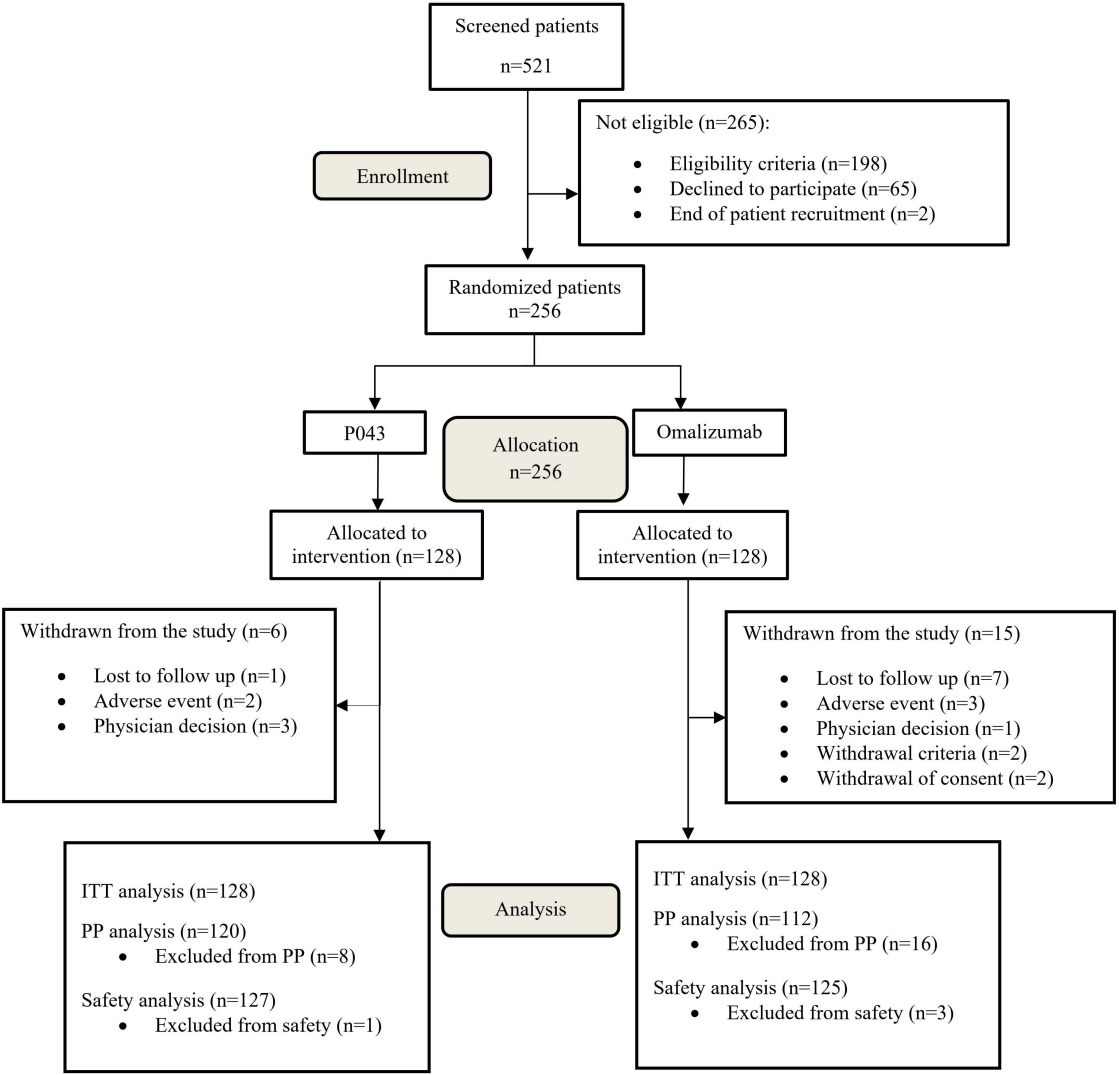
CONSORT flow diagram of study populations. ITT, Intention-to-Treat; PP, Per-Protocol.

The study was initiated on November 2020 and ended on January 2023. A total of 521 participants were screened in seven major cities in Iran, of which 256 were randomized. The CONSORT flow diagram of participants screening and enrolment is available in **Figure 1**. The baseline characteristics and treatment regimens of the study population are presented in **Table 1**.

**Table 1:** Demographics and baseline characteristics of the participants in full analysis set.

Variable	P043 (N = 128)	Omalizumab (N = 128)
Sex (female)	69 (53.9%)	78 (60.9%)
Age (years)	45.88 ± 12.23	47.66 ± 11.88
BMI (kg/m^2^)	28.16 ± 4.89	26.63 ± 4.68
Asthma exacerbation history	3.94 ± 3.08	3.86 ± 2.73
FEV_1_ pre-bronchodilator (predicted %)	69.07 ± 21.33	64.50 ± 22.59
FEV_1_ post-bronchodilator (predicted %)	74.73 ± 21.75	69.73 ± 22.65
ACT score	10.62 ± 2.93	11.09 ± 2.75
Omalizumab dosing		
300 mg every 4 weeks	36 (28.4%)	39 (31.2%)
225 mg every 2 weeks	31 (24.4%)	26 (20.8%)
150 mg every 4 weeks	29 (22.8%)	32 (25.6%)
300 mg every 2 weeks	20 (15.8%)	21 (16.8%)
375 mg every 2 weeks	11 (8.7%)	7 (5.6%)
Fluticasone^α^		
ICS + LABA	14 (10.9%)	13 (10.2%)
ICS + LABA + Other treatment (except OCS)	34 (26.6%)	34 (26.6%)
OCS + ICS + LABA ± Other treatment	22 (17.2%)	22 (17.2%)
Budesonide^α^		
ICS + LABA	8 (6.3%)	9 (7.0%)
ICS + LABA + Other treatment (except OCS)	28 (21.9%)	28 (21.9%)
OCS + ICS + LABA ± Other treatment	22 (17.2%)	22 (17.2%)

ACT, Asthma Control Test; BMI, Body Mass Index; FEV_1_, Forced Expiratory Volume in the first second; ICS, Inhaled Corticosteroid; LABA, Long-Acting Beta Agonists; OCS, Oral Corticosteroids.

Data are presented as numbers (percentage of total participants in the treatment group) or as mean ± SD.

^α^ No patients were enrolled in ICS ± other treatments (except OCS and LABA) stratum.

### 3.1 Primary outcome measure

As reported in **Table 2**, the 28-week rate of PDAEs in the PP population (N=120 in P043 and 112 in omalizumab) was 0.150 (CI: 0.079-0.220) in the P043 group, and 0.190 (CI: 0.110-0.270) in the omalizumab group. The Poisson model in the rate difference calculation was adjusted based on dosing schedule and baseline eosinophils.

**Table 2:** Primary outcome measure analysis.

Outcome	N	P043	N	Omalizumab	Rate difference (95% CI)	P-value
PDAE rate at 28 weeks (PP)	120	0.150 (0.079, 0.220)	112	0.190 (0.110, 0.270)	-0.041 (-0.146, 0.065)ª	0.45
PDAE rate at 28 weeks (ITT)	128	0.212 (0.122, 0.303)	128	0.350 (0.229, 0.471)	-0.138 (-0.286, 0.011)^b^	0.07

CI, Confidence Interval; ITT, Intention-to-Treat; PDAE, Protocol-Defined Asthma Exacerbation; PP, Per-Protocol.

ªPoisson model adjusted based on dosing schedule and baseline eosinophils.

b Negative binomial model adjusted based on dosing schedule and baseline eosinophils.

Similarly, the PDAE rate in ITT population (N=128 in P043 and omalizumab) was 0.21 (CI: 0.12- 0.30) in the P043 group and 0.35 (CI: 0.230-0.47) in the omalizumab group. The negative binomial model in the rate difference calculation was adjusted based on dosing schedule and baseline eosinophils.

**Figure 2:**
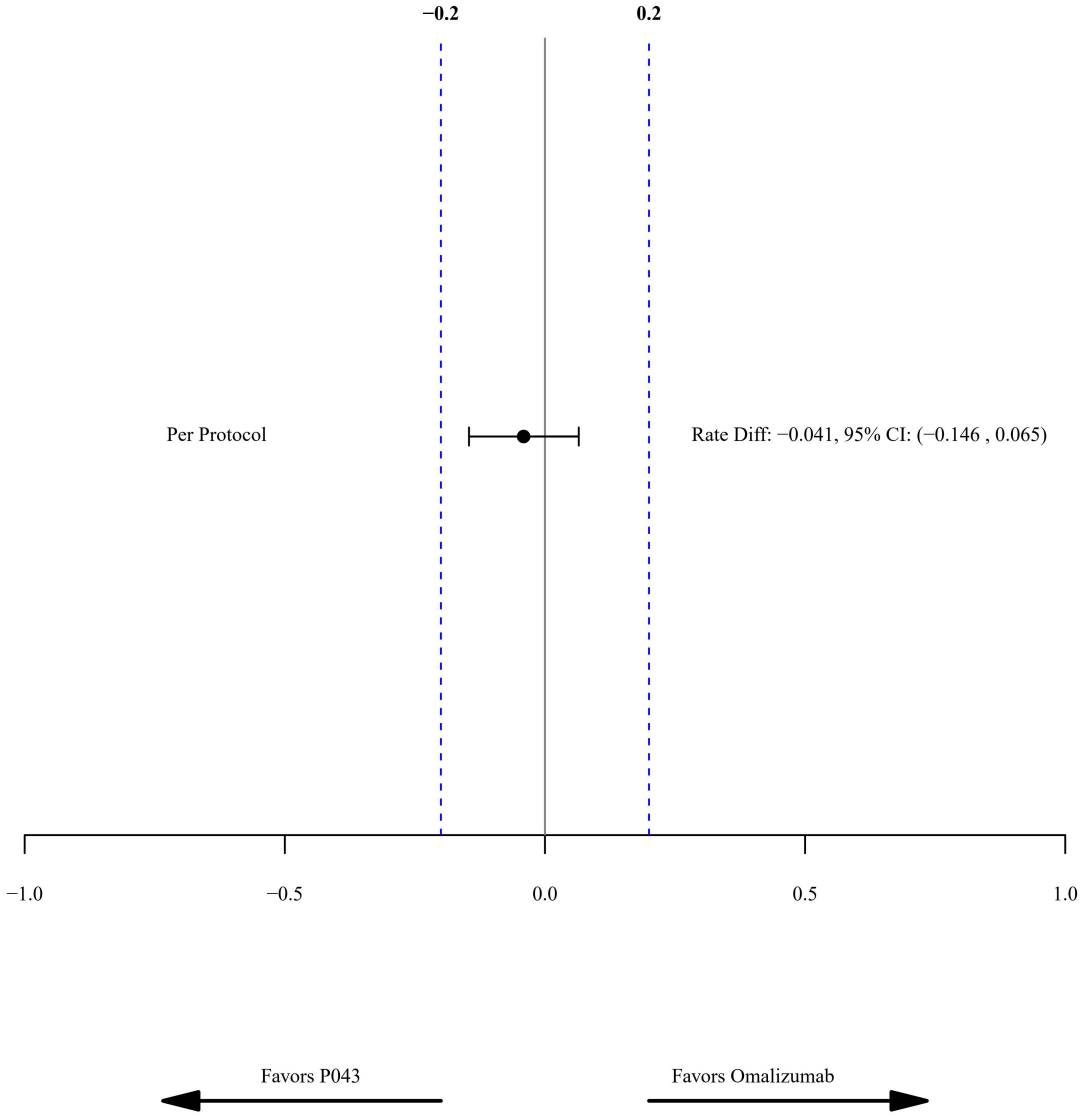
Forest diagram of primary outcome as asthma exacerbations.

The rate difference (95% CI) of the PDAE rate in the PP population was -0.04, with a confidence interval between -0.15 to 0.07. The predefined margin of equivalency was set to 0.2 in the study, as shown in **Figure 2**. The rate difference in the ITT population was -0.14 (CI: -0.29-0.01).

### 3.2 Secondary outcomes measures

#### 3.2.1 FEV_1_ (predicted %); pre, and post-bronchodilator

The means of predicted pre-bronchodilator FEV_1_ were changed from %69.07 ± 21.33 and %64.50 ± 22.59 at the screening to %73.02 ± 20.08 and %74.56 ± 20.61 at the last visit, respectively in the P043 and omalizumab group. Additionally, the means of predicted post-bronchodilator FEV_1_ were elevated from %74.73 ± 21.75 and %69.73 ± 22.65 at the screening to %78.05 ± 20.83 and %81.07 ± 21.01 at the last visit, respectively in the P043 and omalizumab group. The least squares mean (LSM) changes from baseline and the estimated treatment differences are provided in **Table 3**.

**Table 3:** Secondary outcomes measures analysis in the ITT dataset.

Outcome	P043	Omalizumab	P-value
FEV_1_ pre-bronchodilator (predicted %)^a^			
**LSM change from baseline** ** (95% CI)**	5.41 (2.20, 8.63)	7.93 (4.55, 11.30)	0.29
** Estimated treatment** ** difference (95% CI)**	-2.51 (-7.17, 2.15)		
FEV_1_ post-bronchodilator (predicted %)^a^			
** LSM change from baseline** ** (95% CI)**	4.53 (1.14, 7.92)	8.40 (4.85, 11.95)	0.12
** Estimated treatment** ** difference (95% CI)**	-3.87 (-8.79, 1.04)		
ACT score^b^	Parameter Estimate (95% CI)		P-value
** Group**	-0.53 (-1.57, 0.51)		0.32
** Time**	0.63 (0.52, 0.73)		<.001
Time*Group	0.16 (0.01, 0.30)		0.03

ACT, Asthma Control Test; FEV_1_, Forced Expiratory Volume in the first second; LSM, Least Squares Mean.

^a^ANCOVA model adjusted for baseline values.

^b^GEE model (reference group = omalizumab).

#### 3.2.2 ACT scores

**Figure 3:**
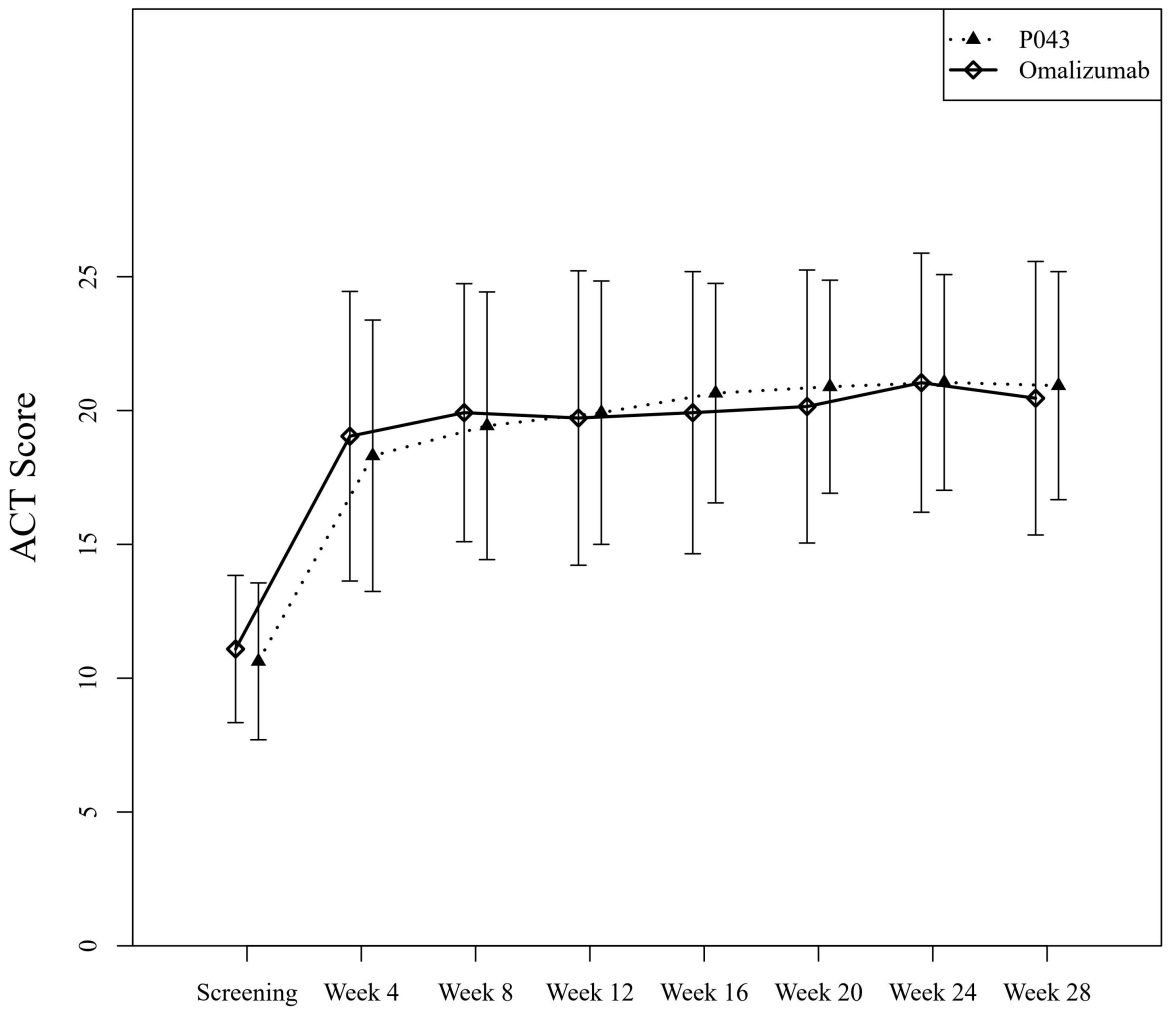
Asthma control evaluated by Asthma Control Test (ACT) scores of self-reported questionnaires.

The mean ± SD of ACT scores at the screening and the last visit were 10.62 ± 2.93 and 20.93 ± 4.26 in the P043 group, and 11.09 ± 2.75 and 20.46 ± 5.11 in the omalizumab group as shown in **Figure 3**. The time-group reciprocal interaction difference of ACT scores in the two groups is shown in **Table 3**.

### 3.3 Safety results

A total of 288 AEs were reported during the study. Seventy-six patients in the P043 group and 71 patients in the omalizumab group reported at least one AE (p-value: 0.62). The incidence of AEs in the SOC of “infections and infestations” (19.7% and 21.6%) and “Respiratory, thoracic and mediastinal disorders” (19.7% and 19.2%) was the highest. The most commonly reported PTs were “dyspnea” (14.2% and 8.8%) and “headache” (12.6% and 8.8%). 45.7% of AEs, and 39.2% of AEs were at least possibly related to study interventions in the P043 group and the omalizumab group, respectively.

Regarding severity, four (3.2%) patients in the P043 group and nine (7.2%) patients in the omalizumab group experienced at least one AE with grade three (P= 0.15). No grade four or five AEs were reported. During the study, 12 SAEs were reported (three SAEs in the P043 group and nine SAEs in the omalizumab group, P=0.07). Additionally, 10 SAEs were related to asthma exacerbations and were analyzed in efficacy data. All 22 reported SAEs resulted in patient hospitalization or prolongation of existing hospitalization and were considered to have an “unlikely” causal relationship to the study intervention by physicians. Among all the mentioned AESIs, injection site reaction (7.9% and 10.4%), hypersensitivity (1.6% and 0.8%), TIA (0.8% and 0.0%) and vasculitis (0.0% and 0.8%) were reported in the P043 group and the omalizumab group, respectively. More details regarding the reported AEs are shown in **Table 4**.

**Table 4:** Safety results.

	P043 (N = 127) *	Omalizumab (N = 125) *
**Number of patients with at least one AE**	76 (59.8%)	71 (56.8%)
	P-value: 0.62	
Common AEs**		
Infections and infestations		
** Nasopharyngitis**	12 (9.5%)	11 (8.8%)
** Corona virus infection**	11 (8.7%)	13 (10.4%)
Respiratory, thoracic and mediastinal disorders		
** Dyspnoea**	18 (14.2%)	11 (8.8%)
Nervous system disorders		
** Headache**	16 (12.6%)	11 (8.8%)
** Dizziness**	7 (5.5%)	1 (0.8%)
General disorders and administration site conditions		
** Injection site reaction**	10 (7.9%)	13 (10.4%)
** Fatigue**	8 (6.3%)	7 (5.6%)
Musculoskeletal and connective tissue disorders		
** Arthralgia**	8 (6.3%)	3 (2.4%)
AEs leading to drug discontinuation		
** Vasculitis**	0 (0.0%)	1 (0.8%)
** Angioedema**	0 (0.0%)	1 (0.8%)
** Drug intolerance ^a^**	2 (1.6%)	0 (0.0%)
** Pneumonia ^b^**	0 (0.0%)	1 (0.8%)

AE, Adverse Event.

Data are presented as number (% of total participants in safety analysis set).

*Safety analysis set.

**Common adverse events were events reported in more than 5% of patients in either group.

^a^Including face edema, dry mouth and influenza like reactions in one patient and diarrhea, vomiting and influenza like reactions in another patient.

^b^This event resulted in intubation of patient.

### 3.4 Immunogenicity

Samples were received at three time-points during the study. Totally, 553 samples were analyzed, of which 295 (53.4%) were from the P043 group and 258 (46.7%) from the omalizumab group. None of the samples tested positive for anti-drug antibodies.

## 4 Discussion

The primary outcome of this study was the rate of asthma exacerbations at 28 weeks, as an indicator of drug efficacy. The incidence rate of exacerbations did not have a statistically significant difference in the P043 group compared to the omalizumab group. The 95% CI for the difference in exacerbation rates did not exceed the predefined margin of 0.2. According to these findings, P043 can be considered equivalent to the reference drug omalizumab in terms of reducing asthma exacerbations over a period of 28 weeks.

The mean annualized observed rate of exacerbations in this study was comparable to the mean annualized rates of exacerbations in prior studies of omalizumab (0.491 in 2304 study, 0.592 in 008C/E study, 0.514 in 009C/E study, and 1.176 in 011 study) (6, 24–26).

In this study, the improvement of lung function was not limited to the decrease of exacerbations. Additionally, ACT scores in both groups increased significantly by the end of the study (P<.001), while the difference in the ACT scores between the two groups was not statistically significant (P=0.32). Improved asthma control was observed in both groups after four weeks of treatment, regardless of their baseline values. However, the time-group reciprocal interaction difference was significant (P=0.03). The means of ACT scores of the omalizumab group until the 12^th^ week were higher than those of the P043 group, while the means of ACT scores of the P043 group were higher than those of the omalizumab group after the 12^th^ week until the 28^th^.

A study by Casale et al. suggests that a significant portion of omalizumab users report improved lung function, despite not experiencing a change in exacerbation rates (27). This highlights the necessity of evaluating both clinical assessment and spirometry measurements as a reflections of lung function.

There was no significant difference between the two groups regarding the changes in the percentage of predicted FEV_1_, before and after bronchodilator use (P= 0.29 and 0.12, respectively). These results are in line with the results of a pooled analysis of five randomized controlled trials (RCTs) that confirms omalizumab would significantly improve FEV_1_ compared to the placebo groups (5). It is worth mentioning that there are studies in which FEV_1_ was not significantly improved after omalizumab treatment, in allergic and non-allergic asthma (7, 28–31). For example, the difference in FEV_1_ at the end of the study between omalizumab and placebo was not significant in the SOLAR study. The baseline mean FEV_1_ in the SOLAR study has been the highest (78.1%) among the main omalizumab studies. The function of FEV_1_ can therefore be viewed as just an additional measure of the efficacy of omalizumab and thus it is concluded that there is some controversy surrounding the effects of omalizumab on spirometry measures. Nevertheless, the results of the present study showed an increasing trend in pre- and post-bronchodilator FEV_1_ in both groups after medication initiation, similar to the results of the five discussed RCTs (5).

Since asthma is a chronic condition, ensuring an acceptable safety profile is imperative for any treatment. The findings of this study indicated that P043 and omalizumab display general comparability in terms of safety aspects. Notably, the overall incidence of AEs (P= 0.62) and SAEs (P= 0.07) had no significant differences between the two groups.

The safety results of this study demonstrate that “infections and infestations” and “Respiratory, thoracic and mediastinal disorders” had the highest incidence among all SOCs in both groups. These findings align with the safety results observed in a study conducted by Nicola A et al. (25).

It is important to note that “injection site reaction” is a known AE associated with omalizumab. According to the safety results of the current study, the incidence of this event was 7.87% and 10.40% in the P043 and omalizumab groups, respectively. Thus, these two products showed almost the same results, which closely mirrors Humbert.et al. study that reported this event at 5.3% in the omalizumab group (24). Furthermore, “dyspnea” and “headache” were the most frequently reported AEs in this study. It is worth mentioning that “dyspnea” was related to asthma symptoms, while the incidence of “headache” was in accordance with omalizumab safety documents (32).

In terms of the seriousness of reported AEs, the study identified that 2.4% of patients in the P043 group and 7.2% of patients in the omalizumab group experienced SAEs, and no significant differences were observed. These results are in line with findings from other studies. For instance, in a study by Nicola A et al., SAEs were reported to be 9.3% in the omalizumab group and 10.5% in the placebo group (25). Additionally, another study by Nicola A et al. focused on evaluating the long-term effectiveness and safety of omalizumab, reporting an incidence of SAEs of 6.9% in adult patients (33). In this study, in line with previous findings from literature reviews, no case of malignancy was reported (34). However, the follow-up period of this study was not long enough to rule out the risk entirely.

This study had some limitations as well. The outbreak of COVID-19 during the study might have caused a decrease in FEV_1_ and ACT scores in both groups due to the mandatory use of face masks. However, Pelaia et al. confirmed that the COVID-19 situation would not alter ACT scores, FEV_1_, and exacerbation rates of patients receiving omalizumab compared with the pre-pandemic era (35). Another limitation of this study is a lack of smoking history recordings in details. Clinical information and data gathered from omalizumab RCTs showed that non-heavy smoking history was not among the confounding factors affecting omalizumab efficacy and therefore this data was not gathered from the participants prior to the enrollment (36–38). Additionally, omalizumab has been associated with improving the symptoms of asthma–chronic obstructive pulmonary disease (COPD) overlap syndrome (ACOS) (39).

In conclusion, the results of the study confirm the equivalency of P043 compared with omalizumab in terms of reducing protocol-defined asthma exacerbations. P043 was also comparable with omalizumab regarding other efficacy and safety measures. The findings of this study suggest that P043 can be used as an omalizumab biosimilar as an add-on treatment for uncontrolled moderate-to-severe allergic asthma patients.

## Data availability statement

The datasets generated and/or analysed during the current study are available from the corresponding author on reasonable request.

## Ethics statement

This study involving humans was approved by Shahid Beheshti University of Medical Sciences (IR.SBMU.NRITLD.REC.1399.133) and Tehran University of Medical Sciences (IR.TUMS.VCR.REC.1399.580) ethics committees. The study was conducted in accordance with the local legislation and institutional requirements. The participants provided their written informed consent to participate in this study.

## Author contributions

MG: Writing – original draft, Writing – review & editing. BG: Writing – original draft, Writing – review & editing. RS: Writing – original draft, Writing – review & editing. MeT: Writing – original draft, Writing – review & editing. MM: Writing – original draft, Writing – review & editing. BA: Writing – original draft, Writing – review & editing. MaT: Writing – original draft, Writing – review & editing. HR: Writing – original draft, Writing – review & editing. MF: Writing – original draft, Writing – review & editing. AK: Writing – original draft, Writing – review & editing. AA: Writing – original draft, Writing – review & editing. FJ: Writing – original draft, Writing – review & editing. SM: Writing – original draft, Writing – review & editing. DA: Writing – original draft, Writing – review & editing. MS: Writing – original draft, Writing – review & editing. ST: Writing – original draft, Writing – review & editing. MH: Writing – original draft, Writing – review & editing. JN: Writing – original draft, Writing – review & editing. FA: Writing – original draft, Writing – review & editing. MF: Writing – original draft, Writing – review & editing. RG: Writing – original draft, Writing – review & editing. AS: Writing – original draft, Writing – review & editing. HK: Conceptualization, Data curation, Formal analysis, Funding acquisition, Investigation, Methodology, Project administration, Resources, Software, Supervision, Validation, Visualization, Writing – original draft, Writing – review & editing. EI: Conceptualization, Data curation, Formal analysis, Investigation, Methodology, Project administration, Resources, Software, Supervision, Validation, Visualization, Writing – original draft, Writing – review & editing.
